# One Disease, Many Genes: Implications for the Treatment of Osteopetroses

**DOI:** 10.3389/fendo.2019.00085

**Published:** 2019-02-19

**Authors:** Sara Penna, Valentina Capo, Eleonora Palagano, Cristina Sobacchi, Anna Villa

**Affiliations:** ^1^San Raffaele Telethon Institute for Gene Therapy (SR-Tiget), San Raffaele Hospital, Milan, Italy; ^2^Translational and Molecular Medicine (DIMET), University of Milano-Bicocca, Monza, Italy; ^3^The National Research Council (CNR) Institute for Genetic and Biomedical Research (IRGB)- CNR-IRGB, Milan Unit, Milan, Italy; ^4^Humanitas Research Hospital, Rozzano, Italy

**Keywords:** bone disease, osteopetrosis, osteoclasts, hematopoietic stem cell transplantation, gene therapy

## Abstract

Osteopetrosis is a condition characterized by increased bone mass due to defects in osteoclast function or formation. In the last decades, the molecular dissection of osteopetrosis has unveiled a plethora of molecular players responsible for different forms of the disease, some of which present also primary neurodegeneration that severely limits the therapy. Hematopoietic stem cell transplantation can cure the majority of them when performed in the first months of life, highlighting the relevance of an early molecular diagnosis. However, clinical management of these patients is constrained by the severity of the disease and lack of a bone marrow niche that may delay immune reconstitution. Based on osteopetrosis genetic heterogeneity and disease severity, personalized therapies are required for patients that are not candidate to bone marrow transplantation. This review briefly describes the genetics of osteopetrosis, its clinical heterogeneity, current therapy and innovative approaches undergoing preclinical evaluation.

## Introduction

The term osteopetrosis derives from the Greek “osteo,” bone, and “petros,” stone, to define a genetically heterogenous group of diseases affecting the skeletal tissue, ranging in severity from benign to fatal in early childhood (1). Osteopetrosis is characterized by increased bone mass due to defective resorption activity or differentiation of osteoclasts (2), causing a disequilibrium of bone turnover, deformities, dental abnormalities and impaired mineral homeostasis, and giving rise to structural fragility that causes frequent fractures. Moreover, osteopetrotic patients are characterized by reduction of marrow cavity, affecting hematologic function; related phenotypes are severe anemia, pancytopenia, frequent infections and hepatosplenomegaly (1, 2) and increased frequency of circulating CD34^+^ cells in the peripheral blood (3). The overly dense cranial nerve foramina lead to impairment of neurologic functions with progressive deafness, blindness and nerve palsies (1, 2). Three different forms of osteopetrosis have been described, based on the pattern of inheritance: autosomal recessive osteopetrosis (ARO), autosomal dominant osteopetrosis (ADO) and X-linked osteopetrosis (2, 4). The only cure for osteopetrosis is allogeneic hematopoietic stem cell transplantation (HSCT), that has greatly improved its outcome overtime (5–7). In this review, we describe the different forms of the disease and therapeutic options, highlighting advances in the setting of safer conditioning regimens and alternative therapies to overcome the limited donor availability.

## Autosomal Recessive Osteopetrosis (ARO)

**Figure 1:**
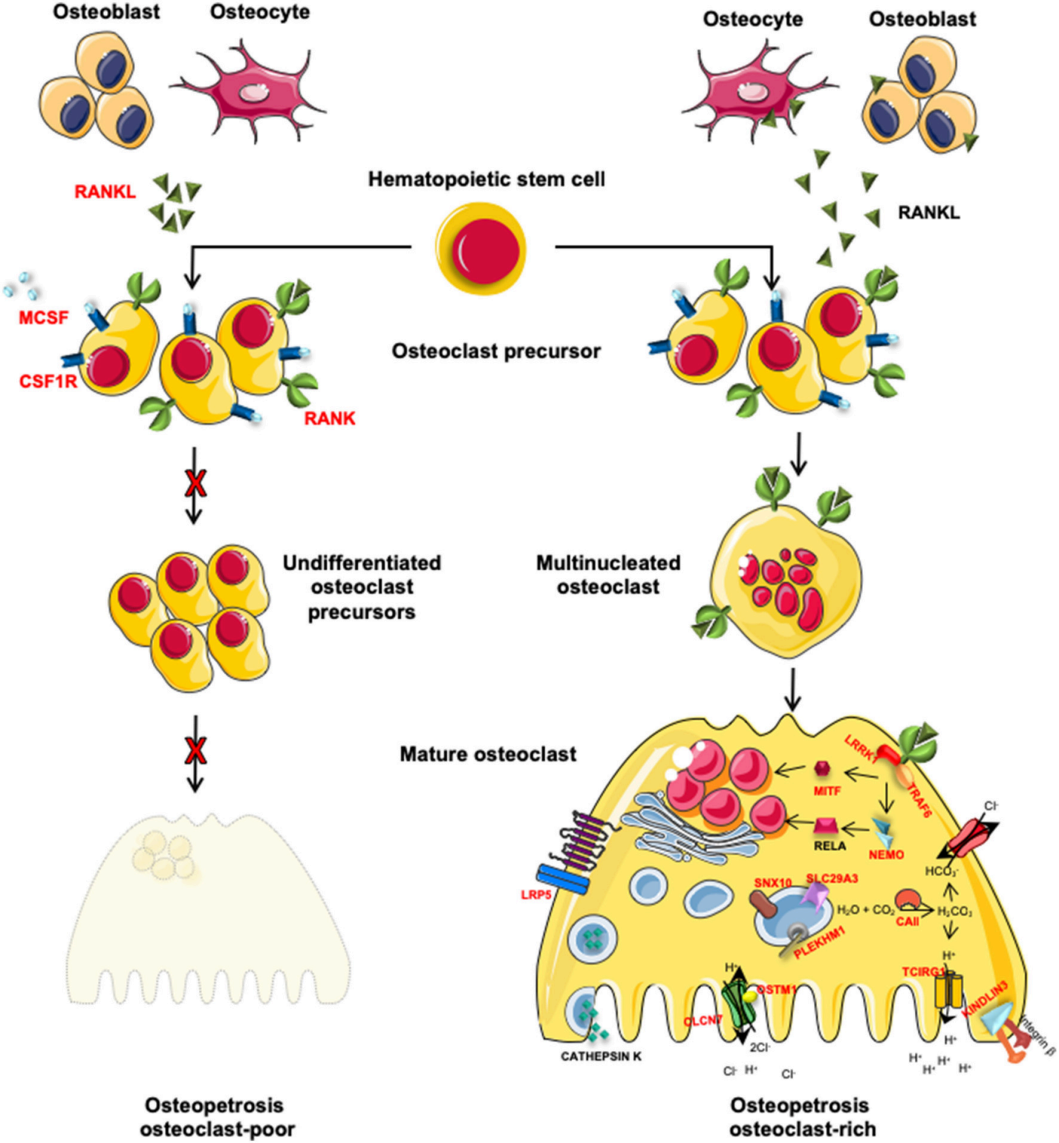
Schematic representation of genes involved in osteoclast-poor and osteoclast-rich osteopetrosis. In red are indicated genes involved in the pathogenesis of ARO. MCSF and RANKL, cytokines secreted by osteoblasts and osteocytes, are necessary for the differentiation of osteoclast precursors into mature and resorbing osteoclasts. When these signals are absent (*TNFSF11* gene mutations) or the pathway is interrupted by the lack of cytokine receptors (*TNFRSF11A* and *CSF1R* gene mutations), osteoclast precursors are not able to differentiate into mature osteoclast causing osteoclast-poor forms of osteopetrosis. Alternatively, if osteopetrosis is caused by mutations in genes encoding for protein necessary for bone resorption, the disease is defined as osteoclast-rich osteopetrosis. On the right of the figure, are indicated genes involved in bone resorption activity with different roles: i.e., acidification of resorption lacunae and pH regulation (*TCIRG1, CLCN7, OSTM1*, and *CAII*), vesicular trafficking and sorting of protein complex to the membrane (*SNX10* and *PLEKHM1*), cytoskeletal rearrangement for ruffle border formation (*FERMT3* and *LRRK1)*. Other molecules involved in different signal transductions, essential for osteoclast functions (*MITF, LRP5*, and *IKBKG*) are reported.

The autosomal recessive form of osteopetrosis (ARO), also known as infantile malignant osteopetrosis (IMO), has an incidence of 1:250000 live births, with higher rates in specific geographic areas because of geographic isolation, high frequency of parental consanguinity or the presence of a founder effect (8). Unless treated with HSCT, ARO is usually fatal within the first 10 years of life (8). Children present with failure to thrive, skull abnormalities (macrocephaly, frontal bossing, choanal stenosis), hydrocephalus, hypocalcemia due to defective calcium mobilization activity of osteoclasts (1) and abnormal tooth eruption with frequent development of dental caries (9). ARO is caused by mutations in different genes that are implicated in osteoclast function (osteoclast-rich osteopetrosis) or differentiation (osteoclast-poor osteopetrosis) (Figure 1).

### Osteoclast-Rich Osteopetrosis

The most frequent form is caused by mutations in the *TCIRG1* (T cell immune regulator 1) gene, accounting for more than 50% of ARO cases. *TCIRG1* encodes for the a3 subunit of V0 complex of the V-ATPase proton pump, mainly expressed by osteoclasts and gastric parietal cells on apical membrane. The V-ATPase pump acidifies the resorption lacuna in the bone for the dissolution of the hydroxyapatite crystals, that form the bone mineral fraction, and the degradation of the matrix (10). The a3 subunit has also been implicated in the interaction between actin cytoskeleton and microtubules, fundamental for the osteoclast ruffled border formation (8, 11). Accordingly, *TCIRG1*-mutated osteoclasts show defective ruffled border and markedly reduced resorptive activity (11, 12). Moreover, the V-ATPase maintains the low pH in the stomach for the dietary Ca^2+^ absorption (13), and, since gastric acidification is also relevant for calcium uptake, this form of osteopetrosis is characterized by rickets or osteomalacia. The second most frequent form of ARO (17% of the cases) is caused by loss of function mutations in the *CLCN7* (chloride voltage-gated channel 7) gene (2, 14). This gene codes a 2Cl^−^/H^+^ antiporter regulated by voltage-gating mechanism, expressed on the osteoclast ruffled border and on the membrane of late endosomes and lysosomes (15). This channel cooperates with the V-ATPase in the acid pH maintenance of the resorption lacuna. CLCN7 is involved in vesicle trafficking in early and recycling endosomes by regulating the luminal Cl^−^ concentration (16). Mutations in the *CLCN7* gene are responsible for a wide spectrum of clinical manifestations. Biallelic mutations cause a very severe form in which bone defects and hematological failure are associated in some patients with primary neurodegeneration, resembling lysosomal storage disease, cerebral atrophy, spasticity, axial hypotonia and peripheral hypertonia (8, 14, 17). Carrier individuals do not show any overt bone phenotype. CLCN7-deficient osteoclasts have been reported to display impaired endolysosomal trafficking (8). In rare intermediate forms of *TCIRG1*- and *CLCN7*-deficient ARO, milder presentation or later onset and slower progression have been recently reported (18–21).

*OSTM1* (osteopetrosis-associated transmembrane protein 1) mutations are reported in 5% of ARO cases (4, 22, 23) and invariably cause osteopetrosis and severe primary neurodegeneration, with a life expectancy lower than 2 years (22, 24–26). OSTM1 has a highly glycosylated N-terminus that has been reported to stabilize CLCN7 protein and to be required, together with its transmembrane region, for CLCN7 Cl^−^/H^+^ transport activity (15). OSTM1 acts also as an E3 ubiquitin ligase for the heterotrimeric G-protein Gαi3 and potentiates WNT canonical signaling by modulating β-catenin/Lef1 interaction (27, 28).

Less than 5% of ARO cases are caused by mutations in the *SNX10* gene, encoding for the sortin nexin 10 protein, one of the major interactors of the V-ATPase. It is involved in the vesicular sorting of the V-ATPase complex from the Golgi network and in its targeting to the ruffled border (8, 29). In the original work, *SNX10*-dependent osteopetrosis was reported to show few and small osteoclasts (30), while in a more recent paper SNX10-deficient osteoclasts were larger and pale at tartrate-resistant acid phosphatase (TRAP) staining (31). Overall, the severity of clinical manifestations is variable (29, 31, 32).

Rare cases of osteoclast-rich osteopetrosis caused by mutations in other genes have also been reported. For example, osteopetrosis caused by carbonic anhydrase II (*CA-II*) deficiency appears in less than one in a million live births and is associated with cerebral calcification and renal tubular acidosis (2, 33). Carbonic anhydrase II enzyme provides protons to the vacuolar proton pump. Since renal defects are more severe than bone abnormalities, *CA-II* deficiency generally is not considered a classic form of ARO (34).

Loss-of-function mutations in the *PLEKHM1* (pleckstrin homology domain–containing family M member 1) gene cause mild osteopetrosis in the *ia* (incisors absent) rat, as well as an intermediate form of human osteopetrosis (35). PLEKHM1 is a cytosolic protein involved in lysosomal trafficking likely acting as an effector of Rab7 (36, 37). Patient-derived *PLEKHM1*-deficient osteoclasts displayed altered morphology and abnormal podosome distribution (35).

Mutations in *FERMT3* (fermitin family member 3) gene have been reported to cause osteopetrosis in association with leukocyte adhesion deficiency type III (LAD III). *FERMT3* gene is expressed in hematopoietic cells and encodes kindlin-3 protein, necessary for integrin signaling and platelet aggregation (38). Patients affected with *FERMT3*-deficiency are characterized by frequent bleeding and recurrent infections (39, 40).

*LRRK1* (leucine-rich repeat kinase 1) gene mutation was found in a single patient affected by osteosclerotic metaphyseal dysplasia, that specifically compromises the methaphyses of long bones, vertebral endplates, costal ends and margin of flat bones (41).

Another mutated gene associated with osteopetrosis is *MITF* (microphtalmia-associated growth factor) that encodes for a transcription factor acting downstream RANK/RANKL pathway (42). *MITF* deficiency is responsible for COMMAD (coloboma, osteopetrosis, microphthalmia, macrocephaly, albinism, and deafness) syndrome in two unrelated patients, suggesting a role for *MITF* in regulating various processes beside bone development and homeostasis (43).

Finally, a homozygous mutation in *C16orf57* has been described in poikiloderma and neutropenia associated with osteopetrosis (44). This gene encodes for a phosphodiesterase responsible for modification and stabilization of the U6 small nuclear RNA, fundamental element of the spliceosome machinery (45), however, its pathophysiologic function in osteoclast still has to be elucidated.

### Osteoclast-Poor Osteopetrosis

The complete absence of osteoclasts is the key feature of the osteoclast-poor form of osteopetrosis (46). Patients are characterized by absence of TRAP-positive osteoclasts in bone biopsies. The defective osteoclastogenesis is caused by either the lack of RANKL (receptor activator of nuclear kappa B ligand) cytokine (2% of all ARO cases) or of its receptor RANK (4.5% of ARO forms) (47–50). RANKL is encoded by the *TNFSF11* gene and the binding to its receptor RANK, encoded by the *TNFRSF11A* gene, determines the activation of the downstream pathway that drives osteoclast differentiation and activation (51). In bone, RANKL is produced mainly by the stromal compartment in physiological condition, while other cell sources are more important in pathological context (52). Recent evidence suggests that RANKL has also an osteogenic role through an autocrine loop in mesenchymal stem cells (53) and through reverse signaling from the osteoclasts to the osteoblasts (54). In addition, in patients RANKL absence leads to a partial defect in T cell proliferation and cytokine production (50), while RANK-deficiency perturbs B cell memory subset and immunoglobulin production (48, 49).

A rare osteoclast-poor form of osteopetrosis, called dysosteosclerosis (DOS), accompanied by red violet macular atrophy, platyspondyly and metaphyseal osteosclerosis, is caused by mutations of the *SLC29A3* (solute carrier family 29 member 3) gene encoding for a lysosomal nucleoside transporter highly expressed in myeloid cells (21, 55). More recently a novel splice-site mutation in the intron 6 of *TNFRSF11A* has been described in one patient indicating *TNFRSF11A* as additional gene responsible for DOS (56).

A recent report described two affected siblings presenting osteopetrosis associated with severe combined immunodeficiency (SCID) caused by a large deletion on chromosome 11 encompassing *RAG1* and *RAG2* genes and the 5′ region of *TRAF6* (TNF receptor-associated factor 6 gene), the most important adaptor for the RANK/RANKL signaling pathway (57).

Lastly, a heterozygous truncating mutation in the *CSF1R* gene, which encodes for MCSF (macrophage colony-stimulating factor) receptor, was reported in the consanguineous parents of two deceased siblings, showing osteopetrosis and brain malformations (58). This mutation could not be assessed in the probands, however based on this report, this genetic variant could be responsible for the disease in this family (59).

## Autosomal Dominant Osteopetrosis

Autosomal Dominant Osteopetrosis (ADO) has an incidence of 1:20,000 live births with clinical onset typically in adolescence or adulthood (4) and cases diagnosed in pediatric age are reported too (18, 60). It is characterized by diffuse osteosclerosis, primarily involving the axial skeleton, and symmetrical defects of the long bones, with no or little modeling defects. ADO form, also known as benign form, is caused by heterozygous missense mutations of the *CLCN7* gene with dominant negative characteristic, in which the mutant subunit is able to dimerize, functionally impairing the protein (12, 17). Patients affected with ADO present a wide range of symptoms: radiographic alterations, frequent atraumatic fractures, osteonecrosis or osteomyelitis, vision and hearing impairment due to cranial nerve compression and occasional bone marrow failure (4, 8, 61). Although *CLCN7* is widely expressed in the body and the biallelic loss of function causes neurodegeneration in some CLCN7-deficient ARO patients, only sporadic cases of cognitive failure have been reported in ADO patients (4, 12).

## X-linked Osteopetrosis

Osteopetrosis caused by mutations of the *IKBKG* (inhibitor of nuclear factor kappa B kinase subunit gamma) gene, located on the X chromosome, occurs as a moderate complication of the OL-EDA-ID syndrome, lymphedema, anhidrotic ectodermal dysplasia and immunodeficiency (hence, the acronym) (62–65). The *IKBKG* gene encodes NEMO, the regulatory subunit of IKK complex, fundamental for the activation of NF-kB transcription factor to induce osteoclastogenesis (62). Consistently, inhibition of NF-kappaB signaling in mouse models of inflammation showed amelioration of osteolysis and inflammation (66). Bone biopsy evaluation in a patient revealed that osteoclasts were present in normal numbers and showed no morphological abnormalities (63). Thus, OL-EDA-ID is classified as an osteoclast-rich osteopetrosis (67).

## Current Therapies and Management of Osteopetrosis

The majority of osteopetrotic forms are caused by osteoclast dysfunction, while a lower proportion of cases are caused by impaired osteoclastogenesis (8). Table 1 summarizes the main clinical features in various forms of osteopetrosis. Since osteoclasts derive from the myeloid lineage, HSCT is the recommended treatment. A successful HSCT allows the engraftment of donor-derived osteoclast precursors, which further differentiate and give rise to functional mature osteoclasts, resulting in bone remodeling and haematopoiesis (9). However, HSCT is contraindicated in patients with primary neurodegenerative disease (Table 1).

**Table 1:** Main clinical features and indications for treatment in osteopetrosis.

**Gene**	**Autosomal recessive osteopetrosis**	**Overall disease severity**	**Hematological defects**	**Visual defects**	**Hypocalcemia**	**Growth retardation**	**Primary neurodegeneration**	**Indication to HSCT**
*TCIRG1*	Osteoclast-rich form	Most often severe	Severe	Mild to severe	Severe	Mild to severe	No	Yes
*ClCN7*	Osteoclast-rich form	Severe to mild	Mild to severe	Mild to severe	Severe	Mild to severe	Yes	To be evaluated based on the severity of CNS involvement
*OSTM1*	Osteoclast-rich form	Severe	Mild to severe	Mild to severe	Moderate	Mild to severe	Yes	No severe CNS involvement
*SNX10*	Osteoclast-rich form	Variable	Severe	Severe	Mild	Mild	No	Yes
*CAII*	Osteoclast-rich form	Moderate	None	Mild	Mild	Moderate	Cerebral calcification	To be evaluated based on cerebral calcification
*PLEKHM1*	Osteoclast-rich form	Mild	None	None	None	None to moderate	No	No mild presentation
*FERMT3*	Osteoclast-rich form	Severe	Severe	Mild	Mild	Mild	No	Yes
*NEMO*	Osteoclast-rich form	Severe	Severe	None	Mild	Moderate	No	Yes
*TNFRSF11A/RANK*	Osteoclast-poor form	Most often severe	Mild	Mild	Mild	Moderate	No	Yes
*TNFSF11/RANKL*	Osteoclast-poor form	Intermediate	Mild	Mild	Mild	Severe	No	No

Since secondary neurological defects are not rescued by transplant, genetic diagnosis and HSCT need to be performed as soon as possible (7, 68, 69). To this end, *in utero* HSC transplantation might represent in the future a therapeutic option as demonstrated by successful preclinical studies performed in the *oc/oc* mouse model (70, 71). Multicentre studies reported that patients undergoing HLA-haploidentical HSCT before the age of 10 months, survived with a full donor engraftment. On the contrary, almost all patients receiving HSCT after the age of 10 months underwent graft rejection or autologous reconstitution, even when an haploidentical donor source was used (7). Taken together, these evidences suggest that the fast diagnosis and timing of treatment, play a fundamental role in the long-term efficacy of HSCT (8). The degree of donor compatibility is another key point to obtain a high rate of 5-years disease-free survival (DFS) after allogenic transplant. Data collected during the years on transplant outcomes, proved that the early diagnosis, the constant monitoring and prompt intervention for the associate comorbidities, the optimization of the donor source in term of HLA-matching and the choice of reduced intensity conditioning regimens allowed higher efficacy and safety of HSCT (9, 69, 72). The most recent report of transplants performed by Ulm and Paris Transplant Units highlighted the improved outcomes of HSCT with 93% of survival using T cell replete matched donor and 80% of survival using T cell depleted haploidentical donor (7). Unrelated cord blood is not recommended because its use is associated to high risk of primary engraftment failure (73). Fludarabine-based conditioning, performed better than the conventional cyclophosphamide-based one, in terms of higher engraftment and reduced toxicity with a higher 5-years DFS. In a selected cohort of 31 patients transplanted from related or unrelated fully matched donors, reduced intensity conditioning (RIC), based on fludarabine, treosulfan and thiotepa with proximal serotherapy dosing using anti-thymocyte globulin, allowed 100% overall survival (69).

The most frequent post-transplant complication is the engraftment failure caused by a delayed hematological reconstitution, due to limited or nearly absent bone marrow space (7) and graft vs. host disease (GvHD) (69). T-cell replete haploidentical graft with the administration of cyclophosphamide after HSCT has been proposed in patients older than 10 months (74). Frequently, transplanted ARO patients showed liver and pulmonary venous-occlusive disease (VOD) (75). Respiratory problems, such as choanal stenosis with upper airway obstructions, capillary leak syndrome, primary pulmonary infections and primary pulmonary hypertension were also frequent. When feasible, the use of a RIC regimen may reduces significantly the incidence of pulmonary hypertension (9, 69).

In addition, central nervous system complications may occur in terms of hydrocephalus, hypocalcaemic convulsions or deterioration of preexisting symptoms. Lastly a recurrent post-transplant risk was the onset of hypercalcemia, that can be treated by the use of Denosumab (76).

## Alternative Treatments and Future Therapies

Despite recent improvement in the HSCT outcome, the availability of HLA-matched donors remains an open issue. For individuals lacking compatible donor, a strategy based on gene therapy (GT) has been proposed. The protocol would exploit the use of genetically modified CD34^+^ cells, isolated from peripheral blood without the need of pharmacological HSC mobilization (3). The efficacy and the feasibility of GT have been studied in the *oc/oc* murine model, to evaluate neonatal transplantation of genetically corrected HSC in the context of *TCIRG1*-dependent osteopetrosis. Retroviral vectors were able to improve bone resorption and survival of *oc/oc* mice (77). Unfortunately, clinical trials in which immunodeficient patients were treated with this type of vector showed the risk of leukemia (78). In recent years, lentiviral vector GT has proven to provide clinical benefit in patients affected by a number of diseases, avoiding the leukemic side effects (79, 80). Moreover, transduction of CD34^+^ cells from the blood of *TCIRG1*-deficient patients with a lentiviral vector achieved the correction of the osteoclast dysfunction *in vitro* (81).

ARO caused by osteoclast extrinsic deficiency, such as *TNFSF11* mutations, requires a different approach. In particular, a replacement therapy has been evaluated at the preclinical level: the product of the *TNFSF11* gene, RANKL cytokine, has been administered pharmacologically to *Tnfsf11* knockout mice, rescuing bone defects and hematopoietic organ architecture (82). Additional strategies could be considered, for example, mesenchymal stem cell (MSC) transplantation to replace the osteoblast precursor population (83); however clinical application still raises doubts and challenges, thus this is far from a mature therapeutic option. The second method exploited the use of biotechnological devices, implanted subcutaneously, to release soluble RANKL and allowing osteoclastogenesis in *Tnfsf11* knockout mice (84). More recently, a promising biomimetic scaffold, seeded with *Tnfsf11* knockout MSC, overexpressing human soluble RANKL after transduction with lentiviral vector has been developed. When implanted subcutaneously, the 3D system was well tolerated and was able to drive the differentiation of TRAP positive cells (85).

Regarding new approaches for the treatment of ADO2, small interfering RNA has been demonstrated to silence specifically the mutated *CLCN7* allele, and to be effective and safe *in vitro* on human cells and *in vivo*, in an ADO2 mouse model (86). Therefore, efforts have been undertaken to move into the clinic (87). Alternatively, the administration of different doses of IFN-γ partially reduced whole-body bone mineral density of ADO2 mice, although further studies for clinical applications are needed (88).

## Conclusions

Genetic dissection of osteopetrosis has unveiled the complex scenario of molecules involved in the pathogenesis of this disease. Early genetic diagnosis is important to establish treatment and thus prevent worsening of the clinical signs. However, despite new molecular techniques have defined ARO molecular complexity, there is the need to further understand their clinical heterogeneity and design novel and suitable cure to these patients. To this end, significant progress has been made in the treatment of ARO thanks to the improvement of novel conditioning regimens and source of donor HSPC, however additional work remains to be done to overcome the limited availability of donors or lack of a therapy for patients carrying RANKL defects or presenting with neurodegenerative osteopetrosis. On this basis, efforts are currently ongoing to further extend the number of molecular players causative of the disease in parallel with the design of novel clinical strategies to be offered as curative treatment for different forms of osteopetrosis.

## Author Contributions

SP, VC, and AV wrote the manuscript. CS and EP critically revised the manuscript and contributed to design the figure.

### Conflict of Interest Statement

The authors declare that the research was conducted in the absence of any commercial or financial relationships that could be construed as a potential conflict of interest.

## Abbreviations

ADO: autosomal dominant osteopetrosis
ARO: autosomal recessive osteopetrosis
DOS: dysosteosclerosis
DSF: disease-free survival
GT: gene therapy
HLA: human leukocyte antigen
HSCT: hematopoietic stem cell transplantation
HSPC: hematopoietic stem progenitor cells
MSC: mesenchymal stem cell
RIC: reduced intensity conditioning
TRAP: tartrate-resistant acid phosphatase.

## References

[1] Wu CC Econs MJ DiMeglio LA Insogna KL Levine MA Orchard PJ. Diagnosis and management of osteopetrosis: consensus guidelines from the osteopetrosis working group. J Clin Endocrinol Metab. (2017) 102:3111–23. 10.1210/jc.2017-01127 28655174

[2] Palagano E Menale C Sobacchi C Villa A. Genetics of osteopetrosis. Curr Osteoporos Rep. (2018) 16:13–25. 10.1007/s11914-018-0415-2 29335834

[3] Steward CG Blair A Moppett J Clarke E Virgo P Lankester A. High peripheral blood progenitor cell counts enable autologous backup before stem cell transplantation for malignant infantile osteopetrosis. Biol Blood Marrow Transplant. (2005) 11:115–21. 10.1016/j.bbmt.2004.11.001 15682072

[4] Teti A Econs MJ. Osteopetroses, emphasizing potential approaches to treatment. Bone (2017) 102:50–9. 10.1016/j.bone.2017.02.002 28167345

[5] Ballet JJ Griscelli C Coutris C Milhaud G Maroteaux P Bone marrow transplantation in osteopetrosis. Lancet (1977) 310:1137 10.1016/S0140-6736(77)90592-X 73050

[6] Coccia PF Krivit W Cervenka J Clawson C Kersey JH Kim TH. Successful bone-marrow transplantation for infantile malignant osteopetrosis. N Engl J Med. (1980) 302:701–8. 10.1056/NEJM198003273021301 6986555

[7] Schulz AS Moshous D Steward CG Villa A Sobacchi C Osteopetrosis. Consensus Guidelines for Diagnosis, Therapy and Follow-Up (2015). Available online at: https://Esid.Org/.2015.654

[8] Sobacchi C Schulz A Coxon FP Villa A Helfrich MH. Osteopetrosis: genetics, treatment and new insights into osteoclast function. Nat Rev Endocrinol. (2013) 9:522–36. 10.1038/nrendo.2013.137 23877423

[9] Natsheh J Drozdinsky G Simanovsky N Lamdan R Erlich O Gorelik N. Improved outcomes of hematopoietic stem cell transplantation in patients with infantile malignant osteopetrosis using fludarabine-based conditioning. Pediatric Blood Cancer (2015) 63:535–40. 10.1002/pbc.25801 26485304

[10] Frattini A Orchard PJ Sobacchi C Giliani S Abinun M Mattsson JP. Defects in TCIRG1 subunit of the vacuolar proton pump are responsible for a subset of human autosomal recessive osteopetrosis. Nat Genet. (2000) 25:343–6. 10.1038/77131 10888887

[11] Nakamura I Takahashi N Udagawa N Moriyama Y Kurokawa T Jimi E. Lack of vacuolar proton ATPase association with the cytoskeleton in osteoclasts of osteosclerotic (Oc/Oc) mice. FEBS Lett. (1997) 401:207–12. 10.1016/S0014-5793(96)01454-8 9013888

[12] Del Fattore A Peruzzi B Rucci N Recchia I Cappariello A Longo M. Clinical, genetic, and cellular analysis of 49 osteopetrotic patients: implications for diagnosis and treatment. J Med Genet. (2006) 43:315–25. 10.1136/jmg.2005.036673 16118345 PMC2563229

[13] Schinke T Schilling AF Baranowsky A Seitz S Marshall RP Linn T. Impaired gastric acidification negatively affects calcium homeostasis and bone mass. Nat Med. (2009) 15:674–81. 10.1038/nm.1963 19448635

[14] Kornak U Kasper D Bösl MR Kaiser E Schweizer M Schulz A. Loss of the ClC-7 chloride channel leads to osteopetrosis in mice and man. Cell (2001) 104:205–15. 10.1016/S0092-8674(01)00206-9 11207362

[15] Leisle L Ludwig CF Wagner FA Jentsch TJ Stauber T ClC-7 is a slowly voltage-gated 2Cl-/1H+-exchanger and requires Ostm1 for transport activity. EMBO J. (2011) 30:2140–52. 10.1038/emboj.2011.137 21527911 PMC3117652

[16] Novarino G Weinert S Rickheit G Jentsch TJ. Endosomal chloride-proton exchange rather than chloride conductance is crucial for renal endocytosis. Science (2010) 328:1398–402. 10.1126/science.1188070 20430975

[17] Pang Q Chi Y Zhao Z Xing X Li M Wang O. Novel mutations of CLCN7 cause autosomal dominant osteopetrosis Type II (ADO-II) and intermediate autosomal recessive osteopetrosis (IARO) in Chinese patients. Osteoporos Int. (2016) 27:1047–55. 10.1007/s00198-015-3320-x 26395888

[18] Pangrazio A Pusch M Caldana E Frattini A Lanino E Tamhankar PM. Molecular and clinical heterogeneity in CLCN7-dependent osteopetrosis: report of 20 novel mutations. Hum Mutat. (2010) 31:1071–80. 10.1002/humu.21167 19953639

[19] Palagano E Blair HC Pangrazio A Tourkova I Strina D Angius A. Buried in the middle but guilty: intronic mutations in the TCIRG1 gene cause human autosomal recessive osteopetrosis. J Bone Miner Res. (2015) 30:1814–21. 10.1002/jbmr.2517 25829125

[20] Sobacchi C Pangrazio A Lopez AG Gomez DP Caldana ME Susani L. As little as needed: the extraordinary case of a mild recessive osteopetrosis owing to a novel splicing hypomorphic mutation in the TCIRG1 gene. J Bone Miner Res. (2014) 29:1646–50. 10.1002/jbmr.2203 24535816 PMC4258090

[21] Howaldt A Nampoothiri S Quell LM Ozden A Fischer-Zirnsak B Collet C. Sclerosing bone dysplasias with hallmarks of dysosteosclerosis in four patients carrying mutations in SLC29A3 and TCIRG1. Bone (2018) 120:495–503. 10.1016/j.bone.2018.12.002 30537558

[22] Chalhoub N Benachenhou N Rajapurohitam V Pata M Ferron M Frattini A. Grey-lethal mutation induces severe malignant autosomal recessive osteopetrosis in mouse and human. Nat Med. (2003) 9:399–406. 10.1038/nm842 12627228

[23] Lange PF Wartosch L Jentsch TJ Fuhrmann JC. ClC-7 requires Ostm1 as a β-subunit to support bone resorption and lysosomal function. Nature (2006) 440:220–3. 10.1038/nature04535 16525474

[24] Pangrazio A Poliani PL Megarbane A Lefranc G Lanino E Di Rocco M. Mutations in OSTM1 (Grey Lethal) define a particularly severe form of autosomal recessive osteopetrosis with neural involvement. J Bone Miner Res. (2006) 21:1098–105. 10.1359/jbmr.060403 16813530

[25] Ott CE Fischer B Schröter P Richter R Gupta N Verma N. Severe neuronopathic autosomal recessive osteopetrosis due to homozygous deletions affecting OSTM1. Bone (2013) 55:292–7. 10.1016/j.bone.2013.04.007 23685543

[26] Overholt KM Rose MJ Joshi S Herman GE Bajwa R Abu-Arja R. Hematopoietic cell transplantation for a child with OSTM1 osteopetrosis. Blood Adv. (2017) 1:279–81. 10.1182/bloodadvances.2016002345 29296943 PMC5727773

[27] Fischer T De Vries L Meerloo T Farquhar MG. Promotion of G Alpha I3 subunit down-regulation by GIPN, a putative E3 ubiquitin ligase that interacts with RGS-GAIP. Proc Natl Acad Sci USA. (2003) 100:8270–5. 10.1073/pnas.1432965100 12826607 PMC166218

[28] Feigin ME Malbon CC. OSTM1 regulates β-Catenin/Lef1 interaction and is required for Wnt/β-catenin signaling. Cell Signal. (2008) 20:949–57. 10.1016/j.cellsig.2008.01.009 18296023 PMC4275117

[29] Pangrazio A Fasth A Sbardellati A Orchard PJ Kasow KA Raza J. SNX10 mutations define a subgroup of human autosomal recessive osteopetrosis with variable clinical severity. J Bone Miner Res. (2013) 28:1041–9. 10.1002/jbmr.1849 23280965

[30] Aker M Rouvinski A Hashavia S Ta-Shma A Shaag A Zenvirt S. An SNX10 mutation causes malignant osteopetrosis of infancy. J Med Genet. (2012) 49:221–6. 10.1136/jmedgenet-2011-100520 22499339

[31] Stattin EL Henning P Klar J McDermott E Stecksen-Blicks C Sandström PE. SNX10 gene mutation leading to osteopetrosis with dysfunctional osteoclasts. Sci Rep. (2017) 7:1–16. 10.1038/s41598-017-02533-2 28592808 PMC5462793

[32] Mégarbané A Pangrazio A Villa A Chouery E Maarawi J Sabbagh S. Homozygous stop mutation in the SNX10 gene in a consanguineous Iraqi boy with osteopetrosis and corpus callosum hypoplasia. Eur J Med Genet. (2013) 56:32–5. 10.1016/j.ejmg.2012.10.010 23123320

[33] Alsharidi A Al-Hamed M Alsuwaida A. Carbonic anhydrase II deficiency: report of a novel mutation. CEN Case Reports (2016) 5:108–12. 10.1007/s13730-015-0205-y 28509178 PMC5411668

[34] Stark Z Savarirayan R. Osteopetrosis. Orphanet J Rare Dis. (2009) 4:5. 10.1186/1750-1172-4-5 19232111 PMC2654865

[35] Van Wesenbeeck L Odgren PR Coxon FP Frattini A Moens P Perdu B. Involvement of PLEKHM1 in osteoclastic vesicular transport and osteopetrosis in incisors absent rats and humans. J Cell Invest. (2007) 117:919–30. 10.1172/JCI30328 17404618 PMC1838941

[36] Fujiwara T Ye S Castro-Gomes T Winchell CG Andrews NW Voth DE. plekhm1/def8/rab7 complex regulates lysosome positioning and bone homeostasis. JCI Insight (2016) 1:e86330. 10.1172/jci.insight.86330 27777970 PMC5070964

[37] Marwaha R Arya SB Jagga D Kaur H Tuli A Sharma M The Rab7 effector PLE KHM1 binds Arl8b to promote cargo traffic to lysosomes. J Cell Biol. (2017) 216:1051–70. 10.1083/jcb.201607085 28325809 PMC5379943

[38] Moser M Nieswandt B Ussar S Pozgajova M Fässler R. Kindlin-3 is essential for integrin activation and platelet aggregation. Nat. Med. (2008) 14:325–30. 10.1038/nm1722. 18278053

[39] McDowall A Svensson L Stanley P Patzak I Chakravarty P Howarth K. Two mutations in the KINDLIN3 gene of a new leukocyte adhesion deficiency III patient reveal distinct effects on leukocyte function *in vitro*. Blood (2010) 115:4834–42. 10.1182/blood-2009-08-238709 20357244

[40] Palagano E Slatter MA Uva P Menale C Villa A Abinun M. Hematopoietic stem cell transplantation corrects osteopetrosis in a child carrying a novel homozygous mutation in the FERMT3 gene. Bone (2017) 97:126–9. 10.1016/j.bone.2017.01.012 28095295

[41] Iida A Xing W Docx MK Nakashima T Wang Z Kimizuka M Identi Fi cation of Biallelic LRRK1 mutations in osteosclerotic metaphyseal dysplasia and evidence for locus heterogeneity. J Med Genet. (2016) 53:568–74. 10.1136/jmedgenet-2016-103756 27055475 PMC5769692

[42] Lu SY Li M Lin YL. Mitf regulates osteoclastogenesis by modulating NFATc1 activity. Exp Cell Res. (2014) 328:32–43. 10.1016/j.yexcr.2014.08.018 25152440 PMC4177974

[43] George A Zand DJ Hufnagel RB Sharma R Sergeev YV Legare JM. Biallelic mutations in MITF cause coloboma, osteopetrosis, microphthalmia, macrocephaly, albinism, and deafness. Am J Human Genet. (2016) 99:1388–94. 10.1016/j.ajhg.2016.11.004 27889061 PMC5142105

[44] Colombo EA Bazan JF Negri G Gervasini C Elcioglu NH Yucelten D. Novel C16orf57 mutations in patients with poikiloderma with neutropenia: bioinformatic analysis of the protein and predicted effects of all reported mutations. Orphanet J Rare Dis. (2012) 7:7. 10.1186/1750-1172-7-7 22269211 PMC3315733

[45] Mroczek S Krwawicz J Kutner J Lazniewski M Kucinski I Ginalski K. C16orf57, a gene mutated in poikiloderma with neutropenia, encodes a putative phosphodiesterase responsible for the U6 SnRNA 3′ end modification. Genes Dev. (2012) 26:1911–25. 10.1101/gad.193169.112 22899009 PMC3435495

[46] Villa A Guerrini MM Cassani B Pangrazio A Sobacchi C. Infantile malignant, autosomal recessive osteopetrosis: the rich and the poor. Calcif Tissue Int. (2009) 84:1–12. 10.1007/s00223-008-9196-4 19082854

[47] Lo Iacono N Pangrazio A Abinun M Bredius R Zecca M Blair HC. RANKL cytokine: from pioneer of the osteoimmunology era to cure for a rare disease. Clin Dev Immunol. (2013) 2013:412768. 10.1155/2013/412768 23762088 PMC3671266

[48] Guerrini MM Sobacchi C Cassani B Abinun M Kilic SS Pangrazio A. Human Osteoclast-Poor Osteopetrosis with Hypogammaglobulinemia Due to TNFRSF11A (RANK) Mutations. Am J Hum Genet. (2008) 83:64–76. 10.1016/j.ajhg.2008.06.015 18606301 PMC2443850

[49] Pangrazio A Cassani B Guerrini MM Crockett JC Marrella V Zammataro L. RANK-dependent autosomal recessive osteopetrosis: characterization of five new cases with novel mutations. J Bone Miner Res. (2012) 27:342–51. 10.1002/jbmr.559 22271396 PMC3306792

[50] Sobacchi C Frattini A Guerrini MM Abinun M Pangrazio A Susani L. Osteoclast-poor human osteopetrosis due to mutations in the gene encoding RANKL. Nat Genet. (2007) 39:960–2. 10.1038/ng2076 17632511

[51] Leibbrandt A Penninger JM. RANK/RANKL: regulators of immune responses and bone physiology. Ann N Y Acad Sci. (2008) 1143:123–50. 10.1196/annals.1443.016 19076348

[52] Liu W Zhang X. Receptor activator of nuclear factor-κB ligand (RANKL)/RANK/osteoprotegerin system in bone and other tissues (Review). Mol Med Rep. (2015) 11:3212–8. 10.2119/molmed.2015.00022 25572286

[53] Schena F Menale C Caci E Diomede L Palagano E Recordati C. Murine Rankl–/– mesenchymal stromal cells display an osteogenic differentiation defect improved by a RANKL-expressing lentiviral vector. Stem Cells (2017) 35:1365–77. 10.1002/stem.2574 28100034

[54] Ikebuchi Y Aoki S Honma M Hayashi M Sugamori Y Khan M. Coupling of bone resorption and formation by RANKL reverse signalling. Nature (2018) 561:195–200. 10.1038/s41586-018-0482-7 30185903

[55] Campeau PM Lu JT Sule G Jiang MM Bae Y Madan S. Whole-exome sequencing identifies mutations in the nucleoside transporter gene SLC29A3 in dysosteosclerosis, a form of osteopetrosis. Human Mol Genet. (2012) 21:4904–9. 10.1093/hmg/dds326 22875837 PMC3607481

[56] Guo L Elcioglu NH Karalar OK Topkar MO Wang Z Sakamoto Y. Dysosteosclerosis is also caused by TNFRSF11A mutation. J Hum Genet. (2018) 769–74. 10.1038/s10038-018-0447-6 29568001

[57] Weisz Hubshman M Basel-Vanagaite L Krauss A Konen O Levy Y Garty BZ. Homozygous deletion of RAG1, RAG2 and 5′ region TRAF6 causes severe immune suppression and atypical osteopetrosis. Clin Genet. (2017) 91:902–7. 10.1111/cge.12916 27808398

[58] Monies D Maddirevula S Kurdi W Alanazy MH Alkhalidi H Al-Owain M. Autozygosity reveals recessive mutations and novel mechanisms in dominant genes: implications in variant interpretation. Genet Med. (2017) 19:1144–50. 10.1038/gim.2017.22 28383543

[59] Dai XM Ryan GR Hapel AJ Dominguez MG Russell RG Kapp S. Targeted disruption of the mouse colony-stimulating factor 1 receptor gene results in osteopetrosis, mononuclear phagocyte deficiency, increased primitive progenitor cell frequencies, and reproductive defects. Blood (2002) 99:111–20. 10.1182/blood.V99.1.111 11756160

[60] Frattini A Pangrazio A Susani L Sobacchi C Mirolo M Abinun M. Chloride channel ClCN7 mutations are responsible for severe recessive, dominant, and intermediate osteopetrosis. J Bone Miner Res. (2009) 18:1740–7. 10.1359/jbmr.2003.18.10.1740 14584882

[61] Waguespack SG Hui SL Dimeglio LA Econs MJ. Autosomal dominant osteopetrosis : clinical severity and natural history of 94 subjects with a chloride channel 7 gene mutation. J Clin Endocrinol Metab. (2007) 92:771–8. 10.1210/jc.2006-1986 17164308

[62] Döffinger R Smahi A Bessia C Geissmann F Feinberg J Durandy A. X-linked anhidrotic ectodermal dysplasia with immunodeficiency is caused by impaired NF-κB signaling. Nat. Genet. (2001) 27:277–85. 10.1038/85837 11242109

[63] Dupuis-Girod S Corradini N Hadj-Rabia S Fournet JC Faivre L Le Deist F. Osteopetrosis, lymphedema, anhidrotic ectodermal dysplasia, and immunodeficiency in a boy and incontinentia pigmenti in his mother. Pediatrics (2002) 109:e97. 10.1542/peds.109.6.e97 12042591

[64] Roberts CM Angus JE Leach IH McDermott EM Walker DA Ravenscroft JC. A novel NEMO gene mutation causing osteopetrosis, lymphoedema, hypohidrotic ectodermal dysplasia and immunodeficiency (OL-HED-ID). Eur J Pediatr. (2010) 169:1403–7. 10.1007/s00431-010-1206-7 20499091

[65] Carlberg VM Lofgren SM Mann JA Austin JP Nolt D Shereck EB. Hypohidrotic ectodermal dysplasia, osteopetrosis, lymphedema, and immunodeficiency in an infant with multiple opportunistic infections. Pediatr Dermatol. (2013) 31:716–21. 10.1111/pde.12103 23405946

[66] Abu-Amer Y Darwech I Otero J. Role of the NF-κB axis in immune modulation of osteoclasts and bone loss. Autoimmunity (2008) 41:204–11. 10.1080/08916930701694543 18365833

[67] Steward CG. Hematopoietic stem cell transplantation for osteopetrosis. Pediatr Clin North Am. (2010) 57:171–80. 10.1016/j.pcl.2009.11.006 20307717

[68] Behfar M Dehghani SS Hosseini AS Jalali A Hamidieh AA Ghavamzadeh A. Non-total body irradiation myeloablative conditioning with intravenous busulfan and cyclophosphamide in hematopoietic stem cell transplantation for malignant infantile osteopetrosis. Pediatr Transplant. (2015) 19:422–7. 10.1111/petr.12476 25879376

[69] Shadur B Zaidman I NaserEddin A Lokshin E Hussein F Oron HC Successful hematopoietic stem cell transplantation for osteopetrosis using reduced intensity conditioning. Pediatric Blood Cancer (2018) 2017:e27010 10.1002/pbc.27010 29469225

[70] Frattini A Blair HC Sacco MG Cerisoli F Faggioli F Catò EM. Rescue of ATPa3-deficient murine malignant osteopetrosis by hematopoietic stem cell transplantation *in utero*. Proc Natl Acad Sci USA. (2005) 102:14629–34. 10.1073/pnas.0507637102 16195375 PMC1253616

[71] Tondelli B Blair HC Guerrini M Patrene KD Cassani B Vezzoni P. Fetal liver cells transplanted *in utero* rescue the osteopetrotic phenotype in the Oc/Oc mouse. Am J Pathol. (2009) 174:727–35. 10.2353/ajpath.2009.080688 19218349 PMC2665735

[72] Orchard PJ Fasth AL Le Rademacher J He W Boelens JJ Horwitz EM. Hematopoietic stem cell transplantation for infantile osteopetrosis. Blood (2015) 126:270–6. 10.1182/blood-2015-01-625541 26012570 PMC4497967

[73] Chiesa R Ruggeri A Paviglianiti A Zecca M Gónzalez-Vicent M Bordon V. Outcomes after unrelated umbilical cord blood transplantation for children with osteopetrosis. Biol Blood Marrow Transplant. (2016) 22:1997–2002. 10.1016/j.bbmt.2016.07.015 27470286

[74] Fuchs EJ. Human leukocyte antigen-haploidentical stem cell transplantation using T-cell-replete bone marrow grafts. Curr Opin Hematol. (2012) 19:440–7. 10.1097/MOH.0b013e32835822dc 22954723

[75] Corbacioglu S Kernan N Lehmann L Brochstein J Revta C Grupp S. Defibrotide for the treatment of hepatic veno-occlusive disease in children after hematopoietic stem cell transplantation. Expert Rev Hematol. (2012) 5:291–302. 10.1586/ehm.12.18 22780209

[76] Shroff R Beringer O Rao K Hofbauer LC Schulz A. Denosumab for post-transplantation hypercalcemia in osteopetrosis. New Engl J Med. (2012) 367:1766–7. 10.1056/NEJMc1206193 23113501

[77] Johansson MK de Vries TJ Schoenmaker T Ehinger M Brun AC Fasth A. Hematopoietic stem cell – targeted neonatal gene therapy reverses lethally progressive osteopetrosis in Oc / Oc mice. Blood (2007) 109:5178–85. 10.1182/blood-2006-12-061382 17332244

[78] Naldini L. *Ex vivo* gene transfer and correction for cell-based therapies. Nat Rev Genet. (2011) 12:301–15. 10.1038/nrg2985 21445084

[79] Sessa M Lorioli L Fumagalli F Acquati S Redaelli D Baldoli C. Lentiviral haemopoietic stem-cell gene therapy in early-onset metachromatic leukodystrophy: an ad-hoc analysis of a non-randomised, open-label, phase 1/2 trial. Lancet (2016) 388:476–87. 10.1016/S0140-6736(16)30374-9 27289174

[80] Thrasher AJ Williams DA. Evolving gene therapy in primary immunodeficiency. Mol Ther. (2017) 25:1132–41. 10.1016/j.ymthe.2017.03.018 28366768 PMC5417846

[81] Moscatelli I Löfvall H Schneider Thudium C Rothe M Montano C Kertész Z. Targeting NSG mice engrafting cells with a clinically applicable lentiviral vector corrects osteoclasts in infantile malignant osteopetrosis. Hum. Gene Ther. (2017) 29:938–49. 10.1089/hum.2017.053. 28726516

[82] Lo Iacono N Blair HC Poliani PL Marrella V Ficara F Cassani B. Osteopetrosis rescue upon RANKL administration to Rankl-/-Mice: a new therapy for human RANKL-dependent ARO. J Bone Miner Res. (2012) 27:2501–10. 10.1002/jbmr.1712 22836362

[83] Bianco P Robey PG (2004). Skeletal stem cells. Handjournal Stem Cells. 2:415–24. 10.1016/B978-012436643-5/50129-2

[84] Cappariello A Paone R Maurizi A Capulli M Rucci N Muraca M. Biotechnological approach for systemic delivery of membrane receptor activator of NF-κB ligand (RANKL) active domain into the circulation. Biomaterials (2015) 46:58–69. 10.1016/j.biomaterials.2014.12.033 25678116 PMC4337851

[85] Menale C Campodoni E Palagano E Mantero S Erreni M Inforzato A MSC-seeded biomimetic scaffolds as a factory of soluble RANKL in rankl-deficient osteopetrosis. Stem Cells Transl Med. (2018) 8:22–34. 10.1002/sctm.18-0085 30184340 PMC6312453

[86] Capulli M Maurizi A Ventura L Rucci N Teti A. Effective small interfering RNA therapy to treat CLCN7-dependent autosomal dominant osteopetrosis Type 2. Mol Ther. (2015) 4:e248. 10.1038/mtna.2015.21 26325626 PMC4877447

[87] Maurizi A Capulli M Patel R Curle A Rucci N Teti A. RNA interference therapy for autosomal dominant osteopetrosis type 2. towards the preclinical development. Bone (2018) 110:343–54. 10.1016/j.bone.2018.02.031 29501587

[88] Alam I Gray AK Acton D Gerard-O'Riley RL Reilly AM Econs MJ Interferon gamma, but not calcitriol improves the osteopetrotic phenotypes in ADO2 mice. J Bone Miner Res. (2015) 30:2005–13. 10.1002/jbmr.2545 25943708

